# Scutellarin Reduces Cerebral Ischemia Reperfusion Injury Involving in Vascular Endothelium Protection and PKG Signal

**DOI:** 10.1007/s13659-021-00322-z

**Published:** 2021-11-08

**Authors:** Ya-Juan Chen, Chen Chen, Meng-Yuan Li, Qing-Qing Li, Xiu-Juan Zhang, Rong Huang, Xing-Wei Zhu, Chun-Yun Bai, Liu-Yi Zhang, Pei-Hua Peng, Wei-Min Yang

**Affiliations:** 1 School of Pharmaceutical Science & Yunnan Key Laboratory of Pharmacology for Natural Products, Kunming Medical University, 1168 Western Chunrong Road, Yuhua Street, Chenggong New City, Kunming, 650500 China; 2 Department of Anesthesiology, The First Affiliated Hospital of Kunming University, Kunming, 650032 China

**Keywords:** Scutellarin, Basilar artery, Vascular endothelium, Ischemia reperfusion, PKG

## Abstract

Flavonoid glycoside scutellarin (SCU) has been widely applied in the treatment of cerebral ischemic diseases in China. In this article, we conducted research on the working mechanisms of SCU in hypoxia reoxygenation (HR) injury of isolated cerebral basilar artery (BA) and erebral ischemia reperfusion (CIR) injury in rat models. In isolated rat BA rings, HR causes endothelial dysfunction (ED) and acetylcholine (ACh) induces endothelium-dependent vasodilation. The myography result showed that SCU (100 µM) was able to significantly improve the endothelium-dependent vasodilation induced by Ach. However, SCU did not affect the ACh-induced relaxation in normal BA. Further studies suggested that SCU (10–1000 µM) dose-dependently induced relaxation in isolated BA rings which were significantly blocked by the cGMP dependent protein kinase (PKG) inhibitor Rp-8-Br-cGMPs (PKGI-rp, 4 µM). Pre-incubation with SCU (500 µM) reversed the impairment of endothelium-dependent vasodilation induced by HR, but the reversing effect was blocked if PKGI-rp (4 µM) was added. The brain slice staining test in rats’ model of middle cerebral artery occlusion (MCAO) induced CIR proved that the administration of SCU (45, 90 mg/kg, iv) significantly reduced the area of cerebral infarction. The Western blot assay result showed that SCU (45 mg/kg, iv) increased brain PKG activity and PKG protein level after CIR surgery. In conclusion, our findings suggested that SCU possesses the ability of protecting brain cells against CIR injury through vascular endothelium protection and PKG signal.

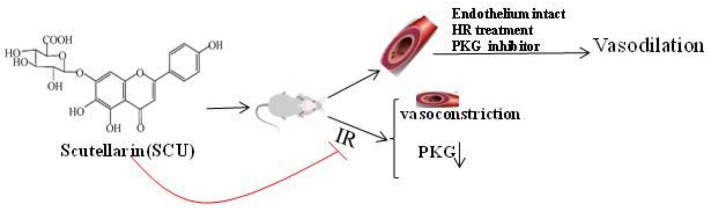

## Introduction

Brain ischemia, also known as cerebral ischemia and cerebrovascular ischemia, is a condition in which there is insufficient blood flow to the brain to meet metabolic demand. The brain is particularly vulnerable to ischemia. This condition may cause symptoms such as alterations in brain metabolism, metabolic rate drops and energy crisis. Severe interruption of brain blood flow can cause the death of vulnerable neurons in multiple brain regions within only 5 min [1]. Therefore, it is crucial to restore the cerebral blood flow in time to prevent permanent brain damage in case of ischemia. The vascular is the most important tissue for blood supply. When cerebral ischemia occurs, the vascular lumen is narrowed lead to vasoconstriction, resulting in a further decrease in blood flow, and aggravating brain damage. Therefore, the vasodilation drugs that can expand blood vessels should be taken to restore the physiological function of brain tissue [2].

Ischemia reperfusion (IR) is defined as the damage that occurs after the restoration of blood supply to ischemic tissue. It can trigger tissue injury caused by various pathophysiological mechanisms. IR directly affects the vascular wall and luminal surface of blood vessels and cause damage such as hemorrhage, capillary plugging, adhesion and infiltration of granulocytes, impaired vascular permeability, and endothelial dysfunction (ED) [3–5]. Endothelial cells which are particularly susceptible to IR injury play an active role in the case of IR-induced organ damage [6]. ED reduces perfusion to ischemia areas and consequently exacerbates tissue injury and subsequent organ damage. Therefore, pharmaceutical interventions that protect the endothelium from IR-induced dysfunction or damage have great therapeutic value in the treatment of cerebral IR injury [7].

It has been reported that acetylcholine (ACh) can increase cerebral blood flow and induce vasodilation in an endothelium-dependent way. However, the responses of cerebral arteries to ACh can be selectively inhibited by hypoxia reoxygenation (HR) [8]. The reason why HR is essential for cerebral IR is that it can trigger various vascular injuries, such as changes in vascular permeability and ED which includes impaired endothelium-dependent vasodilation. It is important to understand how HR mediates ED, and it is necessary to develop medicines and therapeutic methods to reduce or even prevent the risk of cerebral IR injuries.

**Fig. 1:**
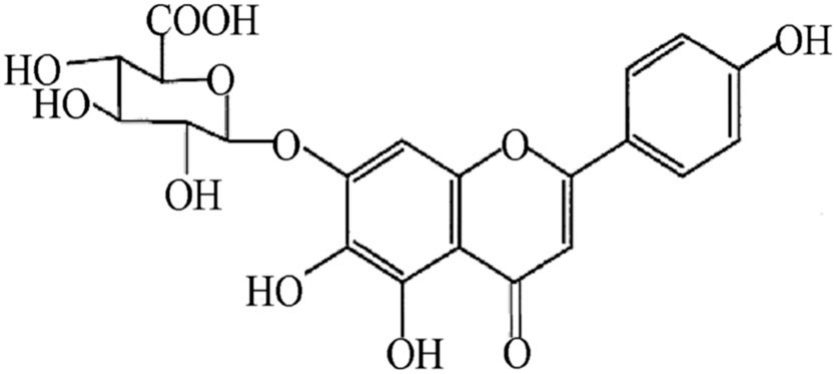
Chemical structure of SCU (4′, 5, 6-trihydroxyflavonoid-7-glucuronide)

Scutellarin (SCU), 4ʹ, 5, 6-trihydroxy flavonoid-7-glucuronide (Fig. 1), is a flavonoid compound found in a traditional Chinese herb *Erigeron breviscapus (vant.)* Hand-Mazz. It has been in clinical use for the treatment of ischemic cardio- and cerebral-vascular diseases in China since the early 1970s [9].SCU is one of the main active ingredients of breviscapine (BRE) which is a flavonoid mixture extracted from Erigeron breviscapus and usually administrated in the form of oral tablet or injection. The general dosage for the treatment of coronary heart disease, angina pectoris, myocardial ischemia and stroke is 50–200 mg/day [10, 11]. In the isolated blood vessels, SCU relaxed the thoracic and abdominal aortas and reduced blood vessel resistance in the isolated blood vessels of rats, and it was able to reduce diastolic blood pressure in rats [12]. Our previous study regarding the working mechanism of SCU has demonstrated that it relaxed the isolated rat thoracic artery rings in an endothelium-dependent manner [13]. Additionally, pharmacological studies showed that SCU can promote vasodilation which is effective against cerebral ischemic injuries. Additionally, SCU can affect ED induced by HR and attenuates cerebral ischemia-induced injuries in rats [14, 15]. However, the exact working mechanism of this effect remains undiscovered.

Many reports suggested that SCU, along with the compounds from the flavonoid family can modulate the tone in the vasculature with endothelium-derived nitric oxide (NO). NO may cause endothelium-dependent and independent relaxations. When NO is generated from L-arginine due to NO Synthase (NOS) action, it activates guanylate cyclase (GC) and produces guanine nuclear glucoside 3',5'-cyclic phosphate (cGMP) which further leads to cGMP-dependent protein kinase (PKG)-mediated vasodilation [16–18]. In particular, the endothelium-derived NOS (eNOS) mediating eNOS-GC-cGMP-PKG pathway plays a key role in maintaining endothelium dependent vascular tone [19, 20]. PKG-I is the strongest vascular tone modulator among the three isoforms of PKG (PKG-I, PKG-I, and PKG-II) [20, 21]. Vasodilation-stimulated phosphoprotein (VASP) was first discovered as a substrate of PKG in human platelets, and it actually exists in various types of cells including the vascular smooth muscle and endothelial cells [22, 23]. The activated PKG phosphorylates VASP which in turn activates downstream ion channels and then triggers endothelium-dependent vasodilation [24]. It’s previously reported in some reports that the eNOS/cGMP/PKG pathways are targets of these flavonoid compounds [15, 25, 26]. Vasodilator stimulated phosphoprotein (VASP) belongs to the Ena/Vasp protein family, is an important PKG-I substrate and an actin regulatory protein. Some studies suggested that phosphorylated VASP at serine 239 (p-VASP) was proven to be a useful monitor agent for the PKG-I activity in intact cells [27]. It has been reported that an analog of SCU, namely baicalin can provide therapeutic effects by regulating the PKG signaling pathway [14]. SCU contains cerebral vascular ED by activating the endothelial PKG pathway [28]. Shi et al. revealed the protective effects of SCU on HR-treated HCMECs and proposed that this effect may be related to the expression changes of the PKG pathway [29]. It has been demonstrated in our previous study that SCU modulated isolated rat coronary artery (CA) vascular tone in an endothelium-dependent manner and it repaired ED induced by HR injury via activating the PKG signal pathway. However, the effect of SCU on cerebral vascular responses and the possible mechanism are still to be discovered.

Our previous studies have shown that the PKG signaling pathway has an effect on the contraction and relaxation of isolated rat CA after HR and SCU antagonizes the changes induced by HR [13, 27, 30]. We also verified the hypothesis that SCU antagonizes ED through PKG signaling pathway in endothelial cell HR model and rat CIR model [31]. However, the previous studies mainly focus on isolated CA and myocardial ischemia reperfusion (MIR) rat model, the effects of SCU on cerebrovascular and its mechanism is not very clear. As SCU has a better effect on cerebral apoplexy than cardiovascular disease in clinical application, in order to better investigate SCU’s mechanism in clinical application in the present investigation we chose to further study the effects of SCU in HR cerebral artery and CIR rats’ model.

In this study, we aimed to investigate the impact of SCU on regulating HR-induced ED in rat basilar artery and explore the role of the PKG pathway in the process. We tested the protein level of PKG-I, VASP and p-VASP in cerebral tissue in the rat models of middle cerebral artery occlusion (MCAO) induced cerebral ischemia reperfusion (CIR). The effects of SCU on cerebral tissue of CIR rats were checked and the role of PKG in the working mechanism of SCU was also studied in a rat model of CIR injury. In conclusion, we planned to discover if SCU could protect the brain against vascular endothelium related CIR injury through the PKG signal pathways.

## Results, Discussion and Conclusion

### SCU-Induced Vasodilation of BA is Dependent on Endothelium

**Fig. 2:**
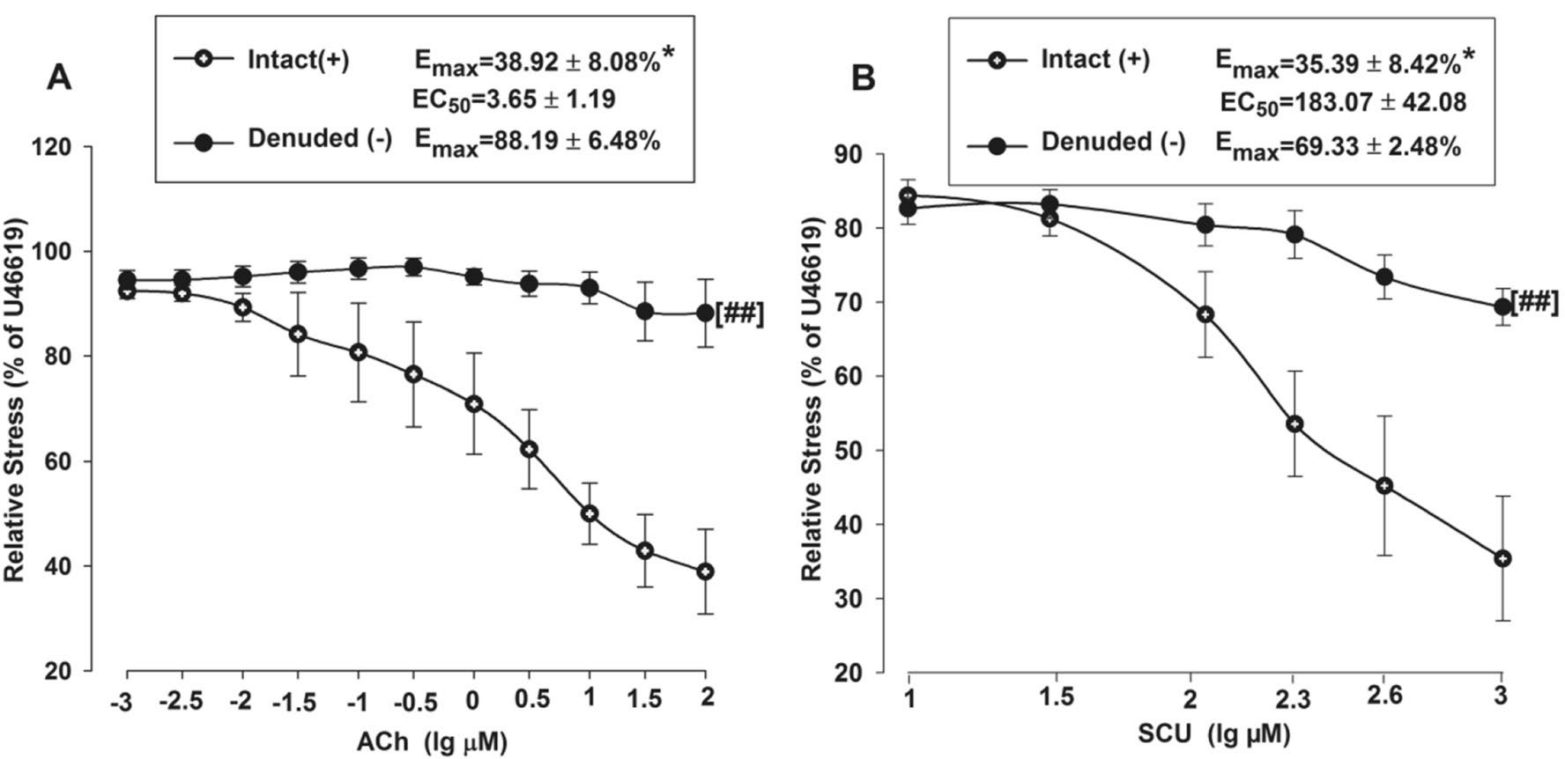
SCU induces endothelium-dependent vasodilation in rat BA. **A** Vasorelaxation induced by ACh (0.001–10 µM) in endothelium-intact and endothelium-denuded BA rings pre-contraction with U46199 (1 µM). **B** Vasorelaxation induced by SCU (10–1000 µM) in endothelium-intact and endothelium-denuded BA rings pre-contraction with U46199 (1 µM). ^*^P < 0.05 vs Denude., t-test of Emax; ^##^P < 0.01 vs Intact, two-way ANOVA test (n = 7–10, Data is presented as mean ± SEM)

The relaxant effects of SCU were examined in BA rings with intact or denuded endothelium in order to investigate if SCU-induced vasodilation is dependent on endothelium. Isolated BA rings were randomly divided into two groups. The endothelium was removed as described in the aforementioned method in the denuded group, while it was kept intact in the control/intact group. ACh was applied to test the denuded model for it can induce endothelium-dependent relaxation. As shown in Fig. 2A, the relative stress was gradually reduced as the ACh concentration increased in the BA intact group, and the changes were minimal in the denuded group. The Emax value was significantly higher in the BA denuded group (88.19%) versus that in the intact BA group (38.92%) (P < 0.05). These results suggested that BA vascular endothelium was successfully denuded. Next, the vasodilation activities of SCU were confirmed at different concentrations (10–1000 µM) (Fig. 2B). These results demonstrated that SCU relaxed endothelium-intact BA rings in a dose-dependent manner. Moreover, the Emax value of SCU (69.33%) in denuded BA was significantly increased than the Emax value (35.39%) in the intact BA (P < 0.05). It suggested that the SCU-induced relaxation was largely endothelium-dependent.

### SCU Does Not Affect Vasodilation Induced by ACh and SNP in Normal BA Rings

**Fig. 3:**
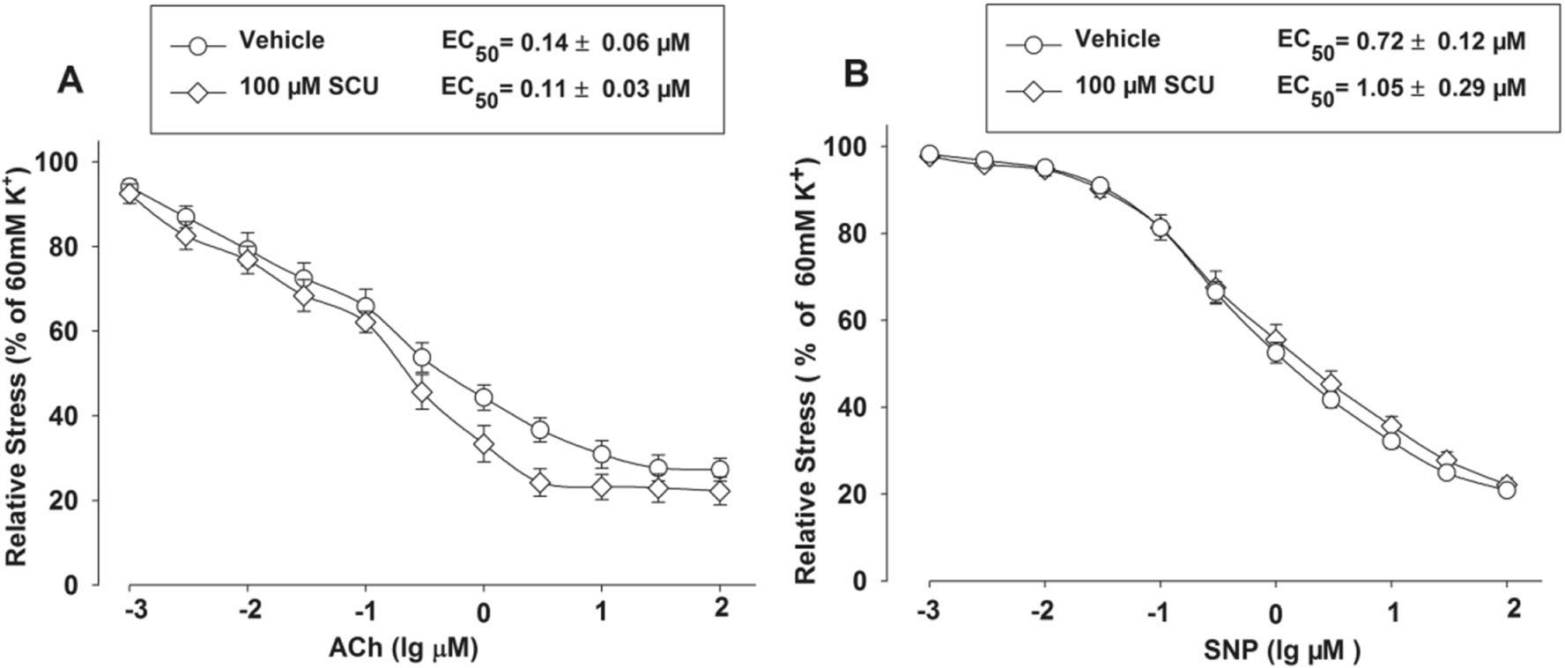
SCU incubation did not affect the relaxation induced by ACh and SNPS in normal BA. The BA segments were randomly divided into three groups and each group contained 9–11 segments. The SCU and ACh groups were pre-incubated with SCU (100 µM) and ACh (0.001–100 µM), respectively. The control group was pretreated with vehicle. The relative stress was calculated afterwards and the data is presented as mean ± SEM

ACh can cause endothelium-dependent vasodilation while SNP can induce endothelium-independent relaxation. We further determined the influence of Ach and SNP on the vasodilation activity of SCU. The normal BA segments were divided into three groups which respectively contained 9–11 segments. Two groups were incubated with SCU (100 µM) before the ACh (0.001–100 µM) or SNP (0.001–100 µM) stimulation. The segments pretreated with the vehicle were used as controls. The results showed that SCU incubation did not significantly alter the ACh-induced endothelium-dependent dilation or the SNP response in intact BA (Fig. 3).

### Effect of SCU on Vasodilation Injury Induced by HR

**Fig. 4:**
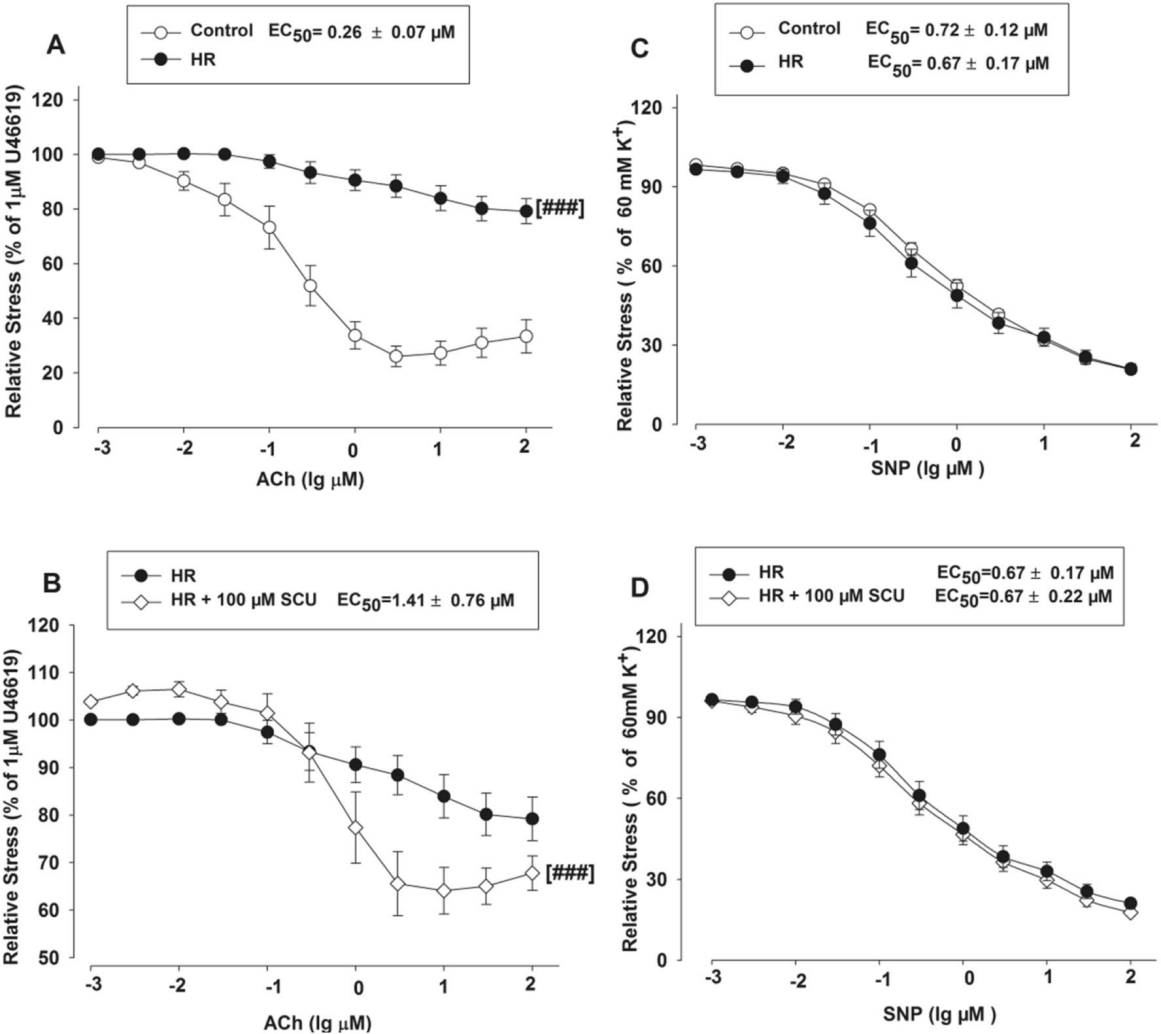
SCU was able to restore ACh-induced endothelium-dependent vasodilation but not SNP-induced endothelium-independent vasodilation in the HR-treated BA rings. The control group was incubated with vehicle without the HR treatment. The HR group was incubated with vehicle after the HR treatment. The HR + 100 µM SCU groups were incubated with SCU (100 µM) after the HR treatment. **A** ACh-induced response decreased in the HR-treated BA. ^###^*P* < 0.001 vs Control, two-way ANOVA test. **B** SCU significantly increased ACh response in HR-treated BA. ^###^*P* < 0.001 vs HR, two-way ANOVA test. **C** SNP-induced dilation was unchanged in the HR-treated BA. **D** SCU did not alter the SNP-induced endothelium-independent dilation in the HR-treated BA. (n = 9 − 11 segments. The data is presented as mean ± SEM)

It has been reported that HR was a crucial factor of cerebral IR injury for it could impair endothelium-dependent vasodilation. We studied the activity of SCU with endothelium-intact or denuded vasodilation in HR-treated BA rings. ACh-induced vasodilation was impaired in the HR treated BA as compared to the BA in the control group without HR treatment (Fig. 4A). This result suggested that HR may impair endothelium function, which in turn blocks endothelium-dependent vasodilation response of BA to ACh. We conducted an experiment to confirm if SCU could reduce ED caused by HR and the method is as follows. The BA segments were preincubated with SCU (100 µM) and followed by the HR treatment before ACh (0.001–100 µM) stimulation. Pre-incubation with SCU (100 µM) significantly reduced the EC_50_ values of ACh in HR-treated BA (Fig. 4B), indicating that SCU was able to restore endothelium-dependent relaxation in HR-treated BA. The results suggested that SCU successfully attenuated HR-induced endothelium injury in rat BA.

We performed another experiment to investigate the activity of SCU on SNP-stimulated endothelium-independent vasodilation by incubating arterial segments with SCU (100 µM) prior to SNP (0.001–100 µM)stimulation in the control and HR-treated BA. Our results showed that SCU did not affect the SNP-induced endothelium-independent dilation in either the control BA or the HR-treated BA (Fig. 4C, D).

### Effects of PKG, NOS, and AC/GC Inhibitors on SCU-Induced Vasodilation

**Fig. 5:**
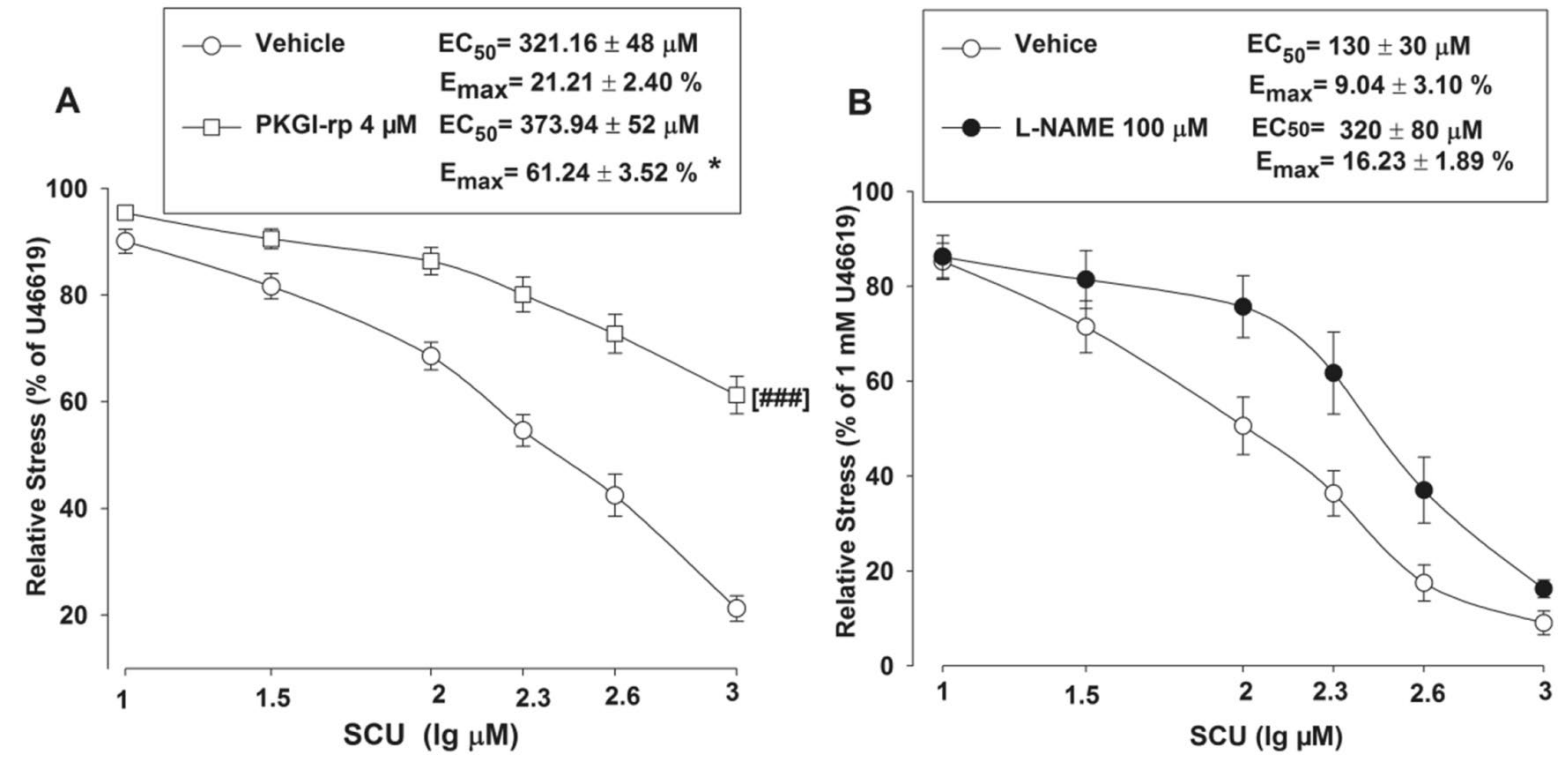
Inhibiting PKG or NOS can reduce SCU-induced vasodilation in intact BA. **A** BA rings were pre-incubated with either the PKG inhibitor PKGI-rp (4 μM) or the vehicle for 15 min, SCU (10–1000 µM) was then accumulatively added into the samples. n = 9–12 segments. **B** The BA rings were pre-incubated with either the NOS inhibitor L-NAME (100 μM) or the vehicle for 15 min, SCU (10–1000 µM) was then accumulatively added into the samples. n = 7–8 segments. The SCU-induced relaxation value is expressed as a percentage of that of the pre-incubation with U46199 (1 µM). Data is presented as mean ± SEM. *P < 0.05, Emax of PKGI-rp vs vehicle. t-test of Emax. ^###^P < 0.001 vs vehicle, two-way ANOVA test (Data are mean ± SEM)

As previously described, SCU could induce vasodilation in intact BA rings. We further investigated into its working mechanism. In this experiment, a total of three inhibitors were tested for their effects on SCU-induced relaxation. Pre-incubation of intact BA with the PKG inhibitor PKGI-rp (4 μM) prior to SCU treatment resulted in a higher EC_50_ value of SCU with a higher E_max_ stress (Fig. 5A), indicating that PKGI-rp significantly inhibited SCU-induced relaxation in BA rings isolated from SD rats.

Additionally, incubation of endothelium-intact BA rings with the NOS inhibitor L-NAME (100 μM) slightly reduced SCU-induced relaxation (Fig. 5B). We further tested if the action of SCU was mediated through AC/GC. Pretreatment of BA rings with the AC inhibitor SQ 22536 (100 µM) and the GC inhibitor ODQ (100 µM) did not significantly inhibit SCU-induced relaxation (data not shown). In short, the results implicated that the SCU-induced endothelium-dependent relaxation was at least partially mediated by the PKG pathway.

### PKG Inhibitor Blocks the Vasorelaxant Effect of SCU in HR-Treated BA

**Fig. 6:**
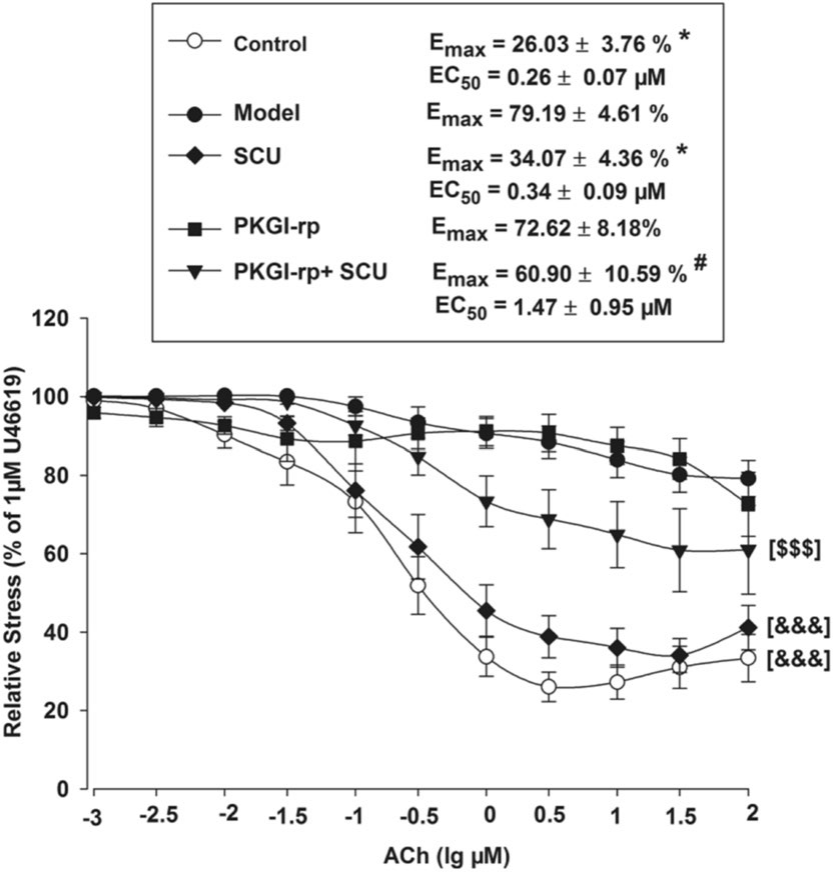
The PKG inhibitor PKGI-rp blocks the protective effect of SCU on ACh-induced endothelium-dependent vasodilation in HR-treated BA. Control: incubated with vehicle without the HR treatment; Model: incubated with vehicle after the HR treatment; SCU, PKGI-rp and PKGI-rp + SCU groups: incubated with SCU (500 μM) or PKGI-rp (4 μM) individually or both PKGI-rp and SCU in combination after HR treatment. ^*^P < 0.05 vs Model, Kruskal–Wallis One Way Analysis of Variance on Ranks of Emax; ^#^*P* < 0.05 vs SCU, Kruskal–Wallis One Way Analysis of Variance on Ranks of Emax; ^&&&^*P* < 0.001 vs Model, two-way ANOVA test; ^$$$^*P* < 0.001 vs SCU, two-way ANOVA test (*n* = 9–12, Data are presented as mean ± SEM)

The incubation test with PKGI-rp (4 μM) prior to SCU (500 μM) treatment significantly reduced, but not completely blocked the protective effect of SCU on ACh-induced endothelium-dependent vasodilation in HR-treated BA (Fig. 6) and the EC_50_ and E_max_ values of ACh were significantly higher. These results suggested that SCU was able to at least partially restore the endothelium-dependent vasodilation in HR-treated BA via the PKG-dependent pathway.

### The Influence of pH Value on the SCU-Induced Vasodilation

**Fig. 7:**
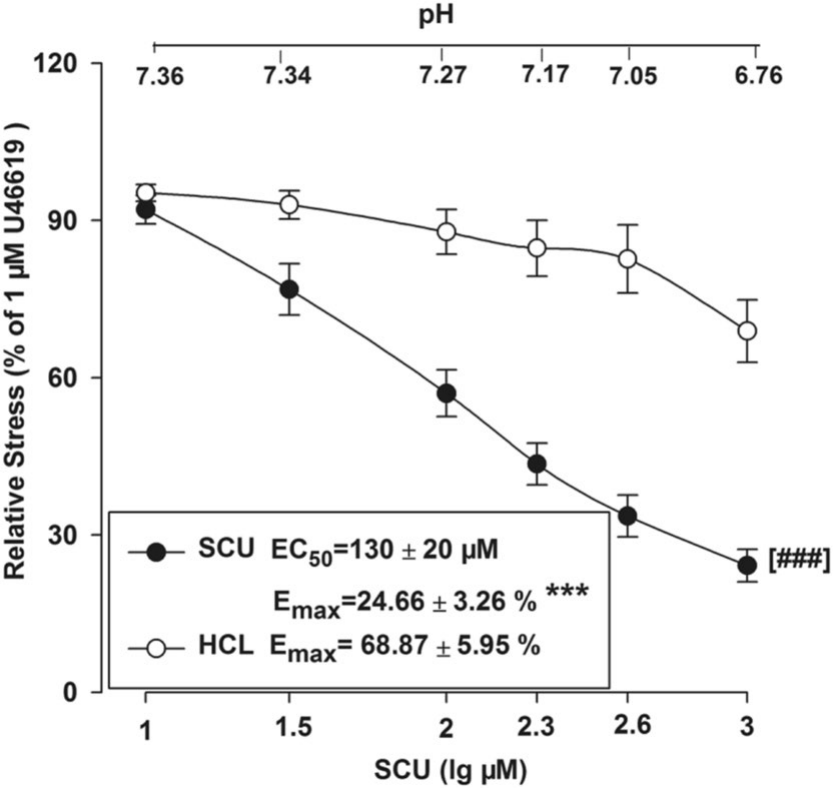
Concentration–response curves of SCU and HCL at the same pH in isolated rat BA rings. SCU groups: incubated with SCU (10–1000 µM). n = 9. HCl groups: HCl (0.36–0.38%) ^***^P < 0.01 vs the corresponding HCL group, *t*-test of E_max_. ^###^*P* < 0.001 vs the corresponding HCl group, two-way ANOVA test (*n* = 10, mean ± SEM)

**Fig. 8:**
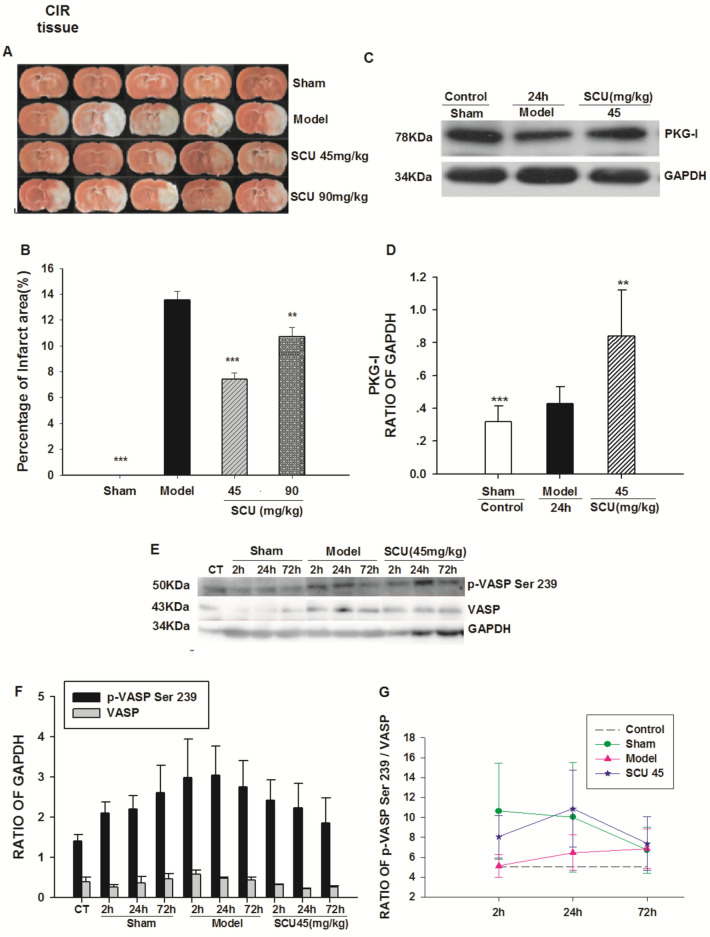
Effect of SCU on PKG activity, and PKG-I protein level in cerebral tissue from CIR rats. **A** Representative images of triphenyl tetrazolium chloride (TTC) staining of ischemic brain slices of CIR (ischemia 60 min/reperfusion 24 h) rats with SCU( 45,90 mg/kg) treatment. **B** Quantification of the corrected cerebral infarct volume in CIR rats with SCU( 45,90 mg/kg) treatment. **C** Representative immunoblot and quantification of PKG-I protein expression in brain tissue of CIR rats with SCU ( 45 mg/kg) treatment. **D** The ratio of PKG-I compared with GAPDH in brain tissue of CIR rats with SCU ( 45 mg / kg) treatment. n = 10 rats in each group. One-way ANOVA followed by SNK test vs. *P < 0.05;**P < 0.01 vs Model;*** P < 0.001 vs Model. **E** The time-effect relationship of SCU with p-VASP Ser 239 protein level in cerebral tissue of CIR rats.Representative immunoblot and quantification of p-VASP Ser 239 and VASP protein expression in brain tissue of CIR rats with SCU ( 45 mg/kg) treatment. **F, G** The ratio of p-VASP compared with VASP in brain tissue of CIR rats with SCU ( 45 mg/kg) treatment. The experimental group: the normal group (Control), the sham operation group (Sham2h, Sham24h, Sham72h), the CIR model group (Model2h, Model24h, Model72h), and the SCU (45 mg/kg) group (SCU2h, SCU24h, SCU72h). All data was analyzed using the One Way Analysis for comparisons. All Pairwise Multiple Comparison Procedures

SCU increases acidity which may further affect the vascular tone. We examined whether an addition of SCU to the saline solution might affect the pH value which may also participate in the observed vascular relaxation activities. The experiment method is as follows. In the tested BA ring sample, HCL was used to create a similar pH to that of SCU in the solution, and the vasodilation effect was recorded. As shown in Fig. 8, HCL slightly dilated the rings but did not show the same level of activity as SCU with generated a significantly different E_max_ value (Fig. 7). In conclusion, pH reduction was proven not to be one of the contributing factors in SCU mediated vasodilation.

### The Influence of SCU on PKG Activities and PKG-I Protein in the Brain of CIR Rats

The percentage of the ischemic area after 24 h of reperfusion in the rat samples was measured to assess the extent of cerebral infarction. As illustrated in Fig. 8 A and B, The infarct area (P < 0.05) was significantly reduced in the SCU group than that in the model group (13.57 + 0.65%). The SCU (45, 90) mg/kg intervention successfully eased the cerebral infarction, especially in the SCU45mg/kg group (7.40 + 0.51%). (Fig. 8) And as shown in Fig. 8C and D, the expression of PKG-I in the brain tissue of CIR rats was increased by SCU (45 mg/kg) compared to that of the Sham group, suggesting that SCU could upregulate the expression of PKG-I.

The effects of SCU (45 mg/kg) on protein expression of p-VASP Ser 239 and VASP in the brain of CIR rats are shown in Fig. 8E. The p-VASP Ser239 / VASP ratio first decreased at 2 h, and then increased at 24 h in the SCU (45 mg/kg) group. The ratio of P-VASP Ser239/VASP in the SCU group (45 mg/kg) was higher than that in the Model group (Fig. 8).

SCU is used in the treatment of hypertensive and cardiovascular diseases traditionally. It has discovered that SCU could lower the blood pressure of rats. SCU is a well-known anti-inflammatory and cardiovascular protective agent which possesses diverse pharmacological activities. SCU is also clinically used to ease some cerebral IR injuries. Given the fact that herbal medicines are increasingly being used in the treatment of vascular diseases, it is essential to understand the working mechanisms of SCU and to provide experimental evidence of its clinical efficacy.

Among the diverse medical applications of SCU, it is mostly used to treat cerebral ischemia for the reason of that it can significantly reduce the volume of cerebral ischemia and the area of cerebral infarction surface. It can also lessen the blood–brain barrier penetration damage. However, the exact working mechanisms of these useful functions are still to be discovered. In this study, we primarily investigated the effects of SCU on vasodilation of BA arteries isolated from rats by monitoring the isometric tension using a wire myograph system. Due to the lack of recognized clinical medicine in the field and the fact that a positive drug is generally not required in the wire myograph system assay, we did not include a positive control in the relevant studies.

Our previous study has focused on the effects of SCU on coronary artery and MIR injury [12, 26, 29]. Although we also investigated the action of SCU in CIR rats (Du et al., 2015), it’s still not very clear with the effects of SCU on cerebral vascular. The main object of this study was the effects of SCU on the contractility and relaxation of isolated rat cerebral arteries after HR and PKG signal pathway.

Both vascular endothelial and smooth muscle cells may be injured from HR exposure. Some studies suggested that the deleterious effect of HR injury was mainly mediated by impaired endothelial function [32–34]. Endothelial cell injuries may occur in the early stage of HR exposure, resulting in the loss of endothelium-dependent vasodilation [35] and deregulated local vascular tone which may ultimately lead to tissue damage. We investigated the effects of SCU on vasodilation in both the intact and endothelium denuded BA rings. The results indicated that SCU dramatically induced relaxation only when the BA ring was intact. In other words, SCU induced vasodilation in an endothelium-dependent manner. In addition, this effect was also dose dependent. Further studies regarding the activities of the endothelium-dependent vasodilator (ACh) showed that HR could cause endothelial damage and reduce the ACh response while SCU could ease the endothelial injury. Meanwhile, SCU did not change the dose–response curve of ACh in the intact sample, indicating that SCU posed no effect on ACh response in intact blood vessels. However, SCU potentially eased the impairment of ACh-induced dilation after HR injury.

SNP is one of the vascular smooth muscle-derived NO providers. It is a non-endothelium-dependent vasodilator that acts directly on the vascular smooth muscle cells by activating the GC induced vasodilation. SCU did not significantly affect the SNP-induced vasodilation in either normal or HR-treated BA. For the reason rat BA was too delicate to be denuded of the endothelium, we couldn’t conduct any experiment to confirm if SCU-induced dilation was inhibited in endothelium-denuded BA in this study. But it was found in our previous studies that SCU-induced vasodilation was reduced by approximately 80% in the tested endothelium-denuded rat aorta. This result indicated that SCU induced vasodilation in an endothelium-dependent manner.

NO plays a key role in blood pressure regulation by triggering NO-dependent vasodilation which is regulated by the NOS/NO/GC/cGMP/PKG pathways in both the endothelium-dependent and the endothelium-independent manners [36]. PKG is expressed in many types of cells, especially in platelets and endothelial cells where its concentrations are usually quite high [21]. PKG signaling is a crucial factor in the regulation of vascular endothelium and smooth muscle relaxation. It has been reported in some studies that the PKG expression level and its activity are closely related to IR injury, atherosclerosis, restenosis, hypertension, and diabetes [30]. Our previous study demonstrated that the role of PKG-Iα might be significant to the protective effects of SCU on ED in the case of MIR injury. The activated PKG phosphorylates VASP which in turn activates downstream ion channels and triggers vascular smooth muscle vasodilation [37]. Previous reports have suggested that the vasodilation effect of baicalin (a flavonoid) was mainly attributed to PKA and the PKG pathways.

In this study, we attempted to research the influence of SCU on the PKG pathways and revealed that the PKG inhibitor PKGI-rp significantly inhibited the protective effect of SCU on endothelium-dependent vasodilation in the HR-treated BA. This finding suggested that SCU might have made an impact on the PKG pathways. To investigate the way how SCU regulates the members of the pathways, we performed the Western blots test to detect the protein levels of PKG, VASP, and p-VASP which were estimated to be the key factors.

One of the downstream targets of the PKGs is the VASP, It is one of the downstream targets of PKG and it is also a protein involved in the control of cytoskeletal dynamics and cell migration [38]. VASP has three phosphorylation sites which are Ser157, Ser239, and Thr278. Serl57 is located in the proline-rich regions (mostly with profilin binding). The determination of P-VASP levels may be used as a novel indicating method for both PKG-I activity and endothelium integrity in human tissues under physiological and pathophysiological conditions. In our recent studies, SCU (45 mg/kg) was tested to be able to increase the expression of PKG-I in the brain tissue of CIR rats. This result suggested that SCU had an influence on the level of PKG-I protein in the brain of CIR rat samples. SCU (45 mg/kg) decreased the expression of p-VASP Ser239, and VASP and increased the ratio of p-VASP Ser239/VASP in 2 h-24 h. The statistical difference could be minimal due to the small number of experiments performed. But it was suggested that SCU could affect the phosphorylation of PKG/PKA in CIR rats. It has also been reported that SCU could significantly reduce the cerebral infarction area and reduce the apoptosis rate of brain cells in rats in the case of ischemia–reperfusion injury. Based on the experimental data acquired in this study, we have reached the conclusion that SCU can reduce cerebral infarction area and reduce cerebral edema caused by ischemia in the IR rat samples.

On the other hand, intracellular and extracellular pH value also has a considerable impact on vascular tone and reactivity of mammalian vascular smooth muscle. However, an acid environment created by HCL had minimal influence on vasodilation. In other words, pH might not be the essential factor regarding the SCU-induced relaxation in HR-treated BA rings.

In conclusion, we demonstrated the following experimental facts. I*n vitro* HR treatment led to impaired endothelium-dependent relaxation in response to ACh in the isolated rat BA rings. SCU attenuated HR-induced ED in an endothelium-dependent and dose-dependent manner in BA arteries. SCU could at least partially induce vasodilation by activating the PKG signaling pathway. SCU was able to reduce the range of cerebral infarction in rats. SCU could ease the decrease of PKG/PKA activity in the brain tissue of CIR rats. These results suggested that the protective effect of SCU on brain tissue is related to the PKG and PKA pathways. This study offers new ideas in the researches regarding the responses of cerebral arteries to HR injury and found a theoretical basis for the application of SCU in the treatment of cerebral IR.

## Experimental Sections

### Animals

Sprague–Dawley (SD) rats (200–250 g) were purchased from the Experimental Animal Center at Kunming Medical University and Xian Communication University. This study was approved by the Animal Care and Use Committee of Kunming Medical University (SYXK2012-2010), and the experiments were conducted according to the standards of Kunming Medical University (SYXK2012-2010).

### Chemicals and Drugs

SCU (yellow powder; purity > 99%; molecular weight, 464.4) was obtained from Kunming Longjin Pharmaceuticals Co. (Kunming, China), its structure is shown as Fig. 1. The NOS inhibitor NO-nitro-L-arginine methylester (L-NAME), the guanyl cyclase (GC) inhibitor 1H-[1,2,4]oxadiazolo[4,3-a]-quinoxalin-1-one (ODQ), the adenyl cyclase (AC) inhibitor 9-(terahydro-2-furanyl)-9H-purin-6-amine (SQ22536), 9,11-dideoxy-11α, 9α-epoxymethano-prostaglandinF2α (U46619), ACh, and sodium nitroprusside (SNP) were purchased from America Sigma Company (Shanghai, China). 3-(N-morpholino) propane sulfonic acid (MOPS) was obtained from Amresco (Shanghai, China). The PKG inhibitor Rp-8-Br-cGMPs (PKGI-rp, purity > 99%) was obtained from Santa Cruz Biotechnology (Shanghai, China). All the salts used in this project were purchased from Biyuntian Co. (Shanghai, China). Rabbit anti-PKG-I and anti-VASP monoclonal antibodies were obtained from Cell Signaling Technology (Hong Kong, China), and Rabbit anti-p-VASP (Ser239) monoclonal antibody was obtained from Millipore (Hong Kong, China).

### Preparation and Equilibration of Isolated BA Rings

Blood vessels were dissected using an integrated wire myograph system (Model 620 M, DMT-Asia Ltd, Shanghai, China) equipped with a Motic SMZ168-TL stereomicroscope. The rats (200–250 g) were sacrificed via intraperitoneal injection of 10% (0.1 mL/100 g) urethane. BA was removed and all the connective tissue was removed by dissection. BA rings (1 mm-long) were picked from cleaned vessels and placed in 4 °C physiological saline solution (PSS) containing 140 mM NaCl, 4.7 mM KCl, 1.6 mM CaCl2, 1.2 mM MgSO4, 1.2 mM MOPS, 1.4 mM Na2HPO4, 0.02 mM EDTA, and 5.6 mM D-glucose. The pH of PSS was pre-adjusted to 7.4 and the temperature was kept at 37 °C.

BA rings were mounted with 60-μm steel wires in separate tissue baths of the wire myograph system to record the tension value. Tissue baths were filled with PSS solution (pH 7.4) aerated with O2 at 37 ± 1 °C. Wash-out was performed by draining and replacing the bathing solution with a syringe. Isometric tension signals were recorded using the Powerlab data acquisition system (AD Instruments Asia, Shanghai, China). The viability of BA rings was measured. The rings which showed less than 10% change of contraction amplitude between two contractions were selected for the following experiments. In some of the experiments where denuded rings were required, the endothelium was mechanically removed by inserting a segment of hair into the lumen and rolling the ring for a few seconds.

### HR Treatment and Evaluation of Endothelium-Dependent and -Independent Vasodilation in BA Rings

HR treatment of isolated BA rings was carried out as previously described [39] with some minor modifications. Blood vessel segments were first incubated in glucose-free PSS pre-aerated with N2 for 60 min, and then transferred into glucose-containing PSS pre-aerated with O2 for 90 min.

ACh (0.001–100 µM) and SNP (0.001 µM-100 µM) was accumulatively added after pre-contraction with KCl (6 × 104 µM) or U46619 (1 µM) and the dose–response curves were recorded to evaluate the influence of the HR treatment on endothelium-dependent and -independent vasodilation in BA rings.

On the other hand, BA rings were incubated with SCU for 15 min before pre-contraction to be evaluated for the influence of SCU on either ACh or SNP induced relaxation. After the HR treatment, the segments were incubated with SCU and the PKG inhibitor for 15 min prior to pre-contraction and their effects on ACh or SNP induced relaxation was assessed afterward.

### The Influence of PKG Inhibitors, NOS, and AC/GC on SCU-Induced Vasodilation

BA rings were pre-contracted with U46619 (1 μM) for 10 min before the SCU treatment at different concentrations ranging from 10 to 1000 μM. Dilation of BA rings in response to SCU was assessed and normalized to 1 µM U46619 in the control group.

Equilibrated BA rings were pre-incubated for 15 min with the PKG inhibitor PKGI-rp (4 µM), the NOS inhibitor L-NAME (1000 μM), the GC inhibitor ODQ (100 µM), and the AC inhibitor SQ22536 (100 µM), respectively. BA rings from the control group were treated with the same volume of the vehicle individually. The rings were then treated with U46619 (1 µM) for 10 min, followed by addition of SCU (10–1000 µM). The dilation responses were recorded and normalized as described above.

### The Influence of pH on SCU-Induced Vasodilation

SCU (10–1000 µM) reduces the pH value, and this effect may cause a change of the vascular tone. In order to verify if SCU can cause dilation via pH modulation in this experiment, we simulated SCU-induced pH changes with an addition of HCL and we recorded the BA stress changes. Firstly, the pH value changes in response to cumulative addition of SCU (10–1000 µM) were measured in the bathing solution using pH and ion electrodes (Mettler-Toledo GmbH, Schwerzenbach, Switzerland). Then we measured the volume of each cumulative addition of HCL (0.36–0.38%) which produced the same pH profile as SCU. Finally, we recorded the stress response when corresponding volumes of HCL were added in isolated rat BA using the Wire Myograph system and compared the response curves of HCL against that of SCU.

### Rat CIR Model and Evaluation of CIR Injury

After overnight fasting, anesthesia was conducted on the rat models using a face mask soaked with isoflurane (2–2.5% mixed with room air). The temperature was kept at 37 ± 1 °C with a heating lamp and an ice bag. Middle cerebral artery occlusion (MCAO) induced focal CIR injury via occlusion of the middle cerebral artery using the improved Longa-Zea method. The animals were incised in the midline of the neck and the soft tissues were retracted. The right common carotid artery (CCA) was identified, and it was followed toward the rostral portion which bifurcated and connected to the external carotid artery (ECA) and the internal carotid artery (ICA). The intraluminal nylon embolus (Beijing Shaodong Biotech Company) was inserted past the ECA stump into the ICA (17–19 mm) until a slight resistance was detected. At this point, the embolus was blocked by the origin of the right MCA for 1 h. After 1 h of ischemia, the embolus was carefully removed to allow MCA reperfusion for 2, 24, and 72 h. The rats in the Sham group suffered the same procedure but did not receive embolus insertion.

The infarct volume was measured after the brains were harvested. The brain samples were sliced into 1-mm coronal sections after 24 h of reperfusion. The cortical infarct volume was measured using 2, 3, 5-triphenyltetrazolium chloride (TTC) according to the manufacturer’s instructions; The sections were stained with 1% TTC in phosphate-buffered saline (pH 7.4) for 10 min at 37 °C. The cross-sectional area of the TTC-unstained region was determined after formalin fixation.

The brain tissues were collected and homogenized on ice. Then lysis buffer was added into the samples. The protein centration was measured with the help of the bicinchoninic acid (BCA) method.

### Western Blot Analysis

Samples of 40 μg total protein were subjected to the western blot analysis. SeeBlue® Plus2 Pre-stained Standard (7μL) from Invitrogen (Carlsbad, CA, USA) was used as the molecular weight marker. The gelelectrophoresis was carried out at 80 V/100 mA for 30 min and then120 V/160 mA for 80 min. The protein samples were then transferred onto the Immobilon PVDF membranes (Immobilon-P, a pore size of 0.45 mm; Millipore Corporation, Billerica, MA) at 15 V/10 mA in 40 min under semi-dry conditions. After the transfer, the membranes were washed in Tris-buffered saline with Tween-20 (TBS-T; 10 mM Tris–HCl, 150 mM NaCl, 0.1% Tween-20, pH 7.6), blocked with 5% skim milk for 1.5 h at room temperature, and then incubated overnight with rabbit anti-PKG-I, anti-VASP, or anti-p-VASP Ser239 monoclonal antibodies (1:1000) and anti-GAPDH antibody (1:5000) at 4 °C. After washing, the membranes were incubated with HRP-conjugated anti-rabbit secondary antibody for 1 h at room temperature. All the membranes were visualized by chemiluminescence detection using an ECL Plus Western Blotting Detection System (GE Healthcare, Buckinghamshire, UK). The protein bands were quantified by densitometric analysis of digitized films using the Scion Image (4.03). The relative intensity was calculated and the result is presented as a percentage of that of the control group.

### Statistics Analyses

The test results are expressed as mean ± SEM. Statistical analyses were performed using the Sigma Stat 3.1 statistical software. EC50 values were calculated according to the dose response curves by nonlinear regression analysis using a Hill algorithm in Sigma Plot 10.0. The Emax values represent the maximal vasodilative responses (minimal relative stresses of pre-contraction). Comparisons between every two groups were preformed using t-tests, and comparisons among three or more groups were conducted using the one-way ANOVA technique. Differences with P < 0.05 were to be considered statistically significant.

## References

[1] Pekna M, Stokowska A, Pekny M (2021). Neurochem. Res..

[2] de With MC, Haug SJ, van der Brigitte HEP, Segal SS (2005). Microcirculation.

[3] Korkmaz-Icöz S, Kocer C, Sayour A, Kraft P, Benker M, Abulizi S, Georgevici A, Brlecic P, Radovits T, Loganathan S, Karck M, Szabó G (2021). Int. J. Mol. Sci..

[4] Shosha E, Fouda A, Lemtalsi T, Haigh S, Fulton D, Ibrahim A, Al-Shabrawey M, Caldwell R, Caldwell R (2021). Mol. Meteb..

[5] Szabó P, Dostal C, Pilz P, Hamza O, Acar E, Watzinger S, Mathew S, Kager G, Hallström S, Podesser B, Kiss A (2021). J. Cardiovasc. Pharmacol. Ther..

[6] Ren W, Huang C, Chu H, Tang Y, Yang X (2021). Curr. Neurovasc. Res..

[7] Zheng C, Song L, Wen J, Li L, Guo Z, Zhou P, Wang C, Li Y, Ma D, Zheng B (2015). J. Cardiovasc. Pharmacol..

[8] VanBenthuysen K, McMurtry I, Horwitz L (1987). J. Clin. Invest..

[9] Wang L, Ma Q (2018). Pharmacol. Ther..

[10] Nestel P (2003). Curr. Opin. Lipidol..

[11] Balzer J, Heiss C, Schroeter H, Brouzos P, Kleinbongard P, Matern S, Lauer T, Rassaf T, Kelm M (2006). J. Cardiovasc. Pharmacol..

[12] Pan Z, Feng T, Shan L, Cai B, Chu W, Niu H, Lu Y, Yang B (2008). Phytother. Res..

[13] Chen Y-J, Wang L, Zhou G-Y, Yu X-L, Zhang Y-H, Hu N, Li Q-Q, Chen C, Qing C, Liu Y-T, Yang W-M (2015). Chin. J. Nat. Med..

[14] Gao J, Chen G, He H, Liu C, Xiong X, Li J, Wang J (2017). Front. Pharmacol..

[15] Yuan Y, Zha H, Rangarajan P, Ling E, Wu C (2014). BMC Neurosci..

[16] Hung Y, Liu Y, Wu B, Yeh J, Hsu J (2021). Front. Physiol..

[17] Lorigo M, Quintaneiro C, Maia C, Breitenfeld L, Cairrao E (2021). Chemosphere.

[18] Abdallah H, Hassan N, El-Halawany A, Mohamed G, Safo M, El-Bassossy H (2020). J. Adv. Res..

[19] Davel C, Wenceslau E, Akamine F, Xavier G, Couto H, Oliveira L (2011). Braz. J. Med. Biol. Res..

[20] Shen Z, Zhang Z, Wang X, Yang K (2018). J. Cell. Biochem..

[21] Chettimada S, Rawat D, Dey N, Kobelja R, Simms Z, Wolin M, Lincoln T, Gupte S (2012). Am. J. Physiol-Lung C..

[22] Wang Z, Wu Y, Zhang S, Zhao Y, Yin X, Wang W, Ma X, Liu H (2019). Nitric Oxide-Biol. Chem..

[23] Warlo E, Arnesen H, Seljeflot I (2019). Thromb. J..

[24] Thorpe R, Stockman S, Williams J, Lincoln T, Pearce W (2013). Am. J. Physiol-Reg..

[25] Chen J, Wang D, Wang F, Shi S, Chen Y, Yang B, Tang Y, Huang C (2017). Peptides.

[26] Gonçalves R, Lugnier C, Keravis T, Lopes M, Fantini F, Schmitt M, Cortes S, Lemos V (2009). Eur. J. Pharmacol..

[27] Shin J, Kweon K, Kim D, Kim P, Jeon T, Maeng S, Sohn N (2018). Am. J. Chin. Med..

[28] Zhang X, Bogunovic D, Payelle-Brogard B, Francois-Newton V, Speer S, Yuan C, Volpi S, Li Z, Sanal O, Mansouri D, Tezcan I, Rice G, Chen C, Mansouri N, Mahdaviani S, Itan Y, Boisson B, Okada S, Zeng L, Wang X, Jiang H, Liu W, Han T, Liu D, Ma T, Wang B, Liu M, Liu J, Wang Q, Yalnizoglu D, Radoshevich L, Uzé G, Gros P, Rozenberg F, Zhang S, Jouanguy E, Bustamante J, García-Sastre A, Abel L, Lebon P, Notarangelo L, Crow Y, Boisson-Dupuis S, Casanova J, Pellegrini S (2015). Nature.

[29] Shi M, Liu Y, Feng L, Cui Y, Chen Y, Wang P, Wu W, Chen C, Liu X, Yang W (2015). Evid.-Based Complement Altern..

[30] Li L, Li L, Chen C, Yang J, Li J, Hu N, Li Y, Zhang D, Guo T, Liu X, Yang W (2015). PLoS ONE.

[31] Du X, Chen C, Zhang M, Cai D, Sun J, Yang J, Hu N, Ma C, Zhang L, Zhang J, Yang W (2015). Evid.-Based Complement. Altern..

[32] Ramírez CM, Zhang X, Bandyopadhyay C, Rotllan N, Sugiyama MG, Aryal B, Liu X, He S, Kraehling JR, Ulrich V, Lin CS, Lasunción M, Li G, Suárez Y, Tellides G, Swirski F, Lee W, Schwartz M, Sessa W, Fernández-Hernando C (2019). Circulation.

[33] Li M, Ke J, Deng Y, Chen C, Huang Y, Bian Y, Guo S, Wu Y, Zhang H, Liu M, Han Y (2021). Front. Pharmacol..

[34] Song Y, Xing H, He Y, Zhang Z, Shi G, Wu S, Liu Y, Harrington E, Sellke F, Feng J (2021). J. Thorac. Cardiovasc. Surg..

[35] Korkmaz B, Buharalioglu K, Sahan-Firat S, Cuez T, Demiryurek AT, Tunctan B (2011). Nitric oxide – Biol. Chem..

[36] Leker R, Shohami E (2002). Brain Res. Rev..

[37] Lin Y, Dai Z, Lin R, Chu K, Chen I, Wu J, Wu B (2010). Phytomedicine.

[38] Michael M, Vehlow A, Navarro C, Krause M (2010). CB..

[39] Bhayadia R, Schmidt B, Melk A, Hömme M (2016). J. Gerontol. A-Biol..

